# Association between fixed orthodontic treatment and pulp stone development: a retrospective study

**DOI:** 10.1007/s00784-026-07247-2

**Published:** 2026-11-11

**Authors:** Anja Ratzmann, Karl-Friedrich Krey, Aike-Siemen Beisser

**Affiliations:** 1 Department of Orthodontics, Center for Dentistry, Oral and Maxillofacial Medicine, University Medicine Greifswald, Fleischmannstrasse 42/ Walther-Rathenau-Str. 42a, 17475 Greifswald, Germany; 2 Private Orthodontic Practice Kieferorthopaedie am Steinweg, Emden, Germany

**Keywords:** Pulp stones, Dental pulp calcification, Orthodontic treatment, Panoramic radiography, Prevalence, Radiographic density

## Abstract

**Objectives:**

This retrospective longitudinal cohort study aimed to evaluate changes in pulp stone prevalence between pretreatment and post-retention panoramic radiographs and to assess potentially associated factors, including sex and active multibracket treatment duration. Additional objectives were to characterize pulp stone distribution by tooth type, jaw, and side and to quantify changes in the radiographic density and pixel-based size of pre-existing pulp stones.

**Materials and methods:**

This study included 199 patients (117 females and 82 males; mean age at T_0_, 12.5 years) who underwent fixed multibracket orthodontic treatment. Panoramic radiographs obtained before orthodontic treatment (T_0_) and after completion of the retention phase (T_1_) were evaluated for radiographically detectable pulp stones in permanent first and second molars. The interval between appliance removal and T_1_ imaging varied among patients and was not recorded separately. Patient- and tooth-level prevalence analyses were performed in the full cohort, whereas changes in radiographic density, expressed as the mean grey value, and pixel-based size of pre-existing pulp stones were quantified using ImageJ in the baseline-positive subgroup (*n* = 38). Statistical analyses included paired and between-group comparisons, correlation analyses, and regression models to evaluate longitudinal changes and potential associations with sex and active multibracket treatment duration.

**Results:**

Patient-level pulp stone prevalence increased significantly from 19.6% at T_0_​​ to 28.6% at T_1_ (p < 0.001). The increase was greater in males than in females (17.1 versus 3.4 percentage points; p = 0.001). At the tooth level, descriptive increases were observed in all evaluated first and second molars. Maxillary molars showed higher prevalence at both time points, whereas the descriptive magnitude of change was broadly comparable between the jaws. Active multibracket treatment duration (mean, 16.6 months) was not significantly associated with an increase in the number of pulp stone-positive molars per patient (p = 0.90). Exploratory quantitative analyses showed positive pooled mean changes in radiographic density and pixel-based size, with substantial variability among measurements. No sex-related differences were identified for either quantitative outcome, while analyses of the association between active multibracket treatment duration and pixel-based size yielded inconsistent results.

**Conclusions:**

Patient-level pulp stone prevalence increased between the pretreatment and post-retention radiographs, with a greater increase observed in males. Maxillary molars showed higher prevalence at both time points. Exploratory quantitative analyses showed positive pooled mean changes in radiographic density and pixel-based size; neither outcome showed sex-related differences, and findings regarding the association between active multibracket treatment duration and pixel-based size were inconsistent. In the absence of an untreated comparison group, these findings describe longitudinal changes within an orthodontically treated cohort but do not establish a causal effect of fixed orthodontic treatment.

**Clinical relevance:**

Patient-level prevalence of radiographically detectable pulp stones was higher at the post-retention assessment than at pretreatment. Because pulp stones may complicate access to the root canal system, their recognition and documentation on clinically indicated radiographs may be relevant if endodontic treatment is required later. The present findings do not justify additional radiographic examinations solely for pulp stone detection.

## Introduction

Multibracket orthodontic appliances are among the most commonly used modalities of fixed orthodontic treatment [1–4]. They enable controlled bodily tooth movement through the application of forces and torques [5], allowing precise correction of complex dental malocclusions. However, fixed orthodontic treatment is also associated with potential risks [6], and previous studies have reported increases in pulp stone occurrence after treatment [7–12]. Multibracket treatment was therefore selected as a clinically relevant model because it is widely used and applies sustained, controlled mechanical forces to multiple teeth over an extended treatment period.

Pulp stones are discrete calcified masses that may occur within the coronal or radicular pulp. Histologically, they have traditionally been classified as true pulp stones, which exhibit dentin-like structures, or false pulp stones, which form through mineralization of degenerating pulpal tissue. Depending on their relationship to the surrounding dentin, they may also be described as free, attached, or embedded [13]. Their etiology is considered multifactorial and remains incompletely understood. Proposed local factors include dental caries, restorative procedures, trauma, periodontal disease, excessive occlusal loading, and aging, while systemic, genetic, and idiopathic factors have also been discussed [13, 14]. Importantly, pulp stones have been identified not only in erupted teeth but also in unerupted and developing teeth that have not been exposed to local conditions within the oral cavity, indicating that local stimuli alone cannot fully explain their formation [15].

Orthodontic tooth movement provides a biologically plausible local stimulus for pulpal changes. Orthodontic force application has been associated with transient alterations in pulpal blood flow as well as cellular and histological responses within the dental pulp [16, 17]. Increased expression of calcitonin gene-related peptide following the application of stronger orthodontic forces further indicates that the pulp responds to mechanical loading [18]. It may be hypothesized that repeated pulpal irritation and subsequent tissue repair promote the deposition of secondary or reparative dentin or other mineralized material, thereby contributing to pulp stone formation or enlargement. However, the available evidence is heterogeneous, and it has not been established that these pulpal responses directly result in pulp stone development [17]. If such processes occur, a longer period of active treatment could entail greater cumulative exposure to orthodontic forces and provide more time for pulpal reparative or mineralization-related changes. Therefore, active treatment duration was examined as a potentially associated factor.

Pulp stones may complicate endodontic treatment by impeding access to the root canal system or, depending on their size and location, causing complete obstruction [13, 14]. Associations between pulp stones and systemic conditions, including nephrolithiasis and aortic calcification, have also been reported [19–22], although their diagnostic significance remains uncertain.

The reported prevalence of pulp stones varies considerably across studies. Older literature has reported values ranging from approximately 8% to 90% [23, 24]. This variability may reflect differences in study populations, geographic settings, age distributions, evaluated tooth types and pulpal regions, units of analysis, and diagnostic methods [23, 25–28]. Comparative studies have demonstrated differences in pulp stone detection among panoramic radiography, periapical radiography, and cone-beam computed tomography (CBCT), reflecting differences in spatial resolution, anatomical representation, and the potential for superimposition-related misinterpretation [26, 27]. Prevalence estimates obtained using different imaging modalities are therefore not directly comparable.

Previous studies have examined pulp stone occurrence in association with orthodontic treatment but have primarily focused on their binary presence or prevalence. Comparability is limited by differences in study design, imaging modality, evaluated tooth types, and outcome definitions [7–12]. More recent studies have expanded this evidence by comparing clear aligners with fixed appliances and extraction with non-extraction treatment protocols. These studies reported increases in pulp stone occurrence but found no significant associations with appliance type, active treatment duration, or extraction status [29, 30]. Nevertheless, potential associations with patient- and treatment-related factors remain uncertain, while paired quantitative changes in the radiographic characteristics of pre-existing pulp stones have not been systematically investigated.

Therefore, this retrospective study aimed to assess longitudinal changes in pulp stone prevalence in permanent first and second molars of patients undergoing fixed orthodontic treatment. Prevalence was evaluated at both patient and tooth levels, and pulp stone distribution was characterized by tooth type, jaw, and side. Sex-related differences in patient-level prevalence change were examined, while sex and active multibracket treatment duration were additionally evaluated as potentially associated with an increase in the number of pulp stone-positive molars per patient. In addition, changes in the radiographic density and pixel-based size of pre-existing pulp stones were assessed between T_0_ and T_1_. Radiographic density was quantified using the mean grey value (MGV) as an indirect imaging-based surrogate for calcification degree rather than a direct measure of mineral content. The paired quantitative assessment of radiographic density and pixel-based size represents a principal novel component of the present study. We hypothesized that patient-level pulp stone prevalence would be higher at T_1_ than at T_0_. Paired quantitative changes in radiographic density and pixel-based size and their potential associations with sex and active multibracket treatment duration were evaluated exploratorily without prespecified directional hypotheses.

## Materials and methods

### Study population

This retrospective single-center study was conducted using clinical records and panoramic radiographs from a private orthodontic practice in Emden, Germany. All included patients were treated at this practice, and all radiographs were acquired at the same center. Information on individual nationality or ethnic background was not available in the retrospective records.

In the present study, the occurrence of pulp stones in permanent first and second molars was analyzed using panoramic radiographs (orthopantomograms, OPTs) obtained from an initial pool of 360 patients who underwent multibracket orthodontic treatment. The radiographic observation period extended from the earliest T_0_ radiograph acquired in 2015 to the latest T_1_ radiograph acquired in 2020. All records were individually screened according to the predefined inclusion and exclusion criteria. A total of 161 patients were excluded because one or more eligibility criteria were not met, resulting in a final cohort of 199 patients (82 males and 117 females), corresponding to 1,592 evaluated molars. Reason-specific exclusion counts were not retained in the retrospective screening documentation and could therefore not be reconstructed. At the start of orthodontic treatment (T_0_), the patients had a mean age of 12.47 ± 1.12 years (range, 11–17 years). The mean duration of active multibracket treatment was 16.64 ± 3.91 months (range, 10–29 months). Age at T_1_ could not be calculated reliably because the complete interval between T_0_ and T_1_, including the variable retention period, was not recorded separately.

The presence of permanent dentition and the eruption status of the second molars were verified retrospectively by reviewing the clinical patient records, treatment documentation, and available clinical images. Second molars were considered sufficiently erupted when orthodontic attachments could be bonded and the pulp chamber could be reliably assessed on the panoramic radiographs.

All radiographic images were obtained as part of routine orthodontic diagnostics before the initiation of orthodontic treatment (T_0_) and as part of the final diagnostic assessment after completion of the retention phase (T_1_). The T_1_ radiographs were therefore not obtained immediately after removal of the fixed multibracket appliances. Consequently, no additional radiographic exposures beyond standard clinical care were required.

The inclusion criteria were as follows:

- Availability of evaluable OPTs at T_0_ (pretreatment) and T_1_ (post-retention assessment).
- Presence of a permanent dentition, with all second molars sufficiently erupted to permit bonding of orthodontic attachments and reliable radiographic assessment.
- No history of tooth extractions.
- No combined orthodontic-orthognathic surgical treatment.
- Absence of dental caries in teeth examined.

The exclusion criteria were as follows:

- Extensive dental restorations or prosthetic reconstructions.
- Endodontically treated teeth.
- Disorders of dentin or enamel formation.
- Presence of systemic diseases.

No formal a priori sample size calculation was performed. The study sample was derived from an initial pool of 360 patient records assembled for retrospective screening within the radiographic observation period. After application of the predefined eligibility criteria, 199 eligible and evaluable patient records comprised the final cohort. The final sample size was therefore determined by the number of eligible records within the initial screening pool.

**Fig. 1:**
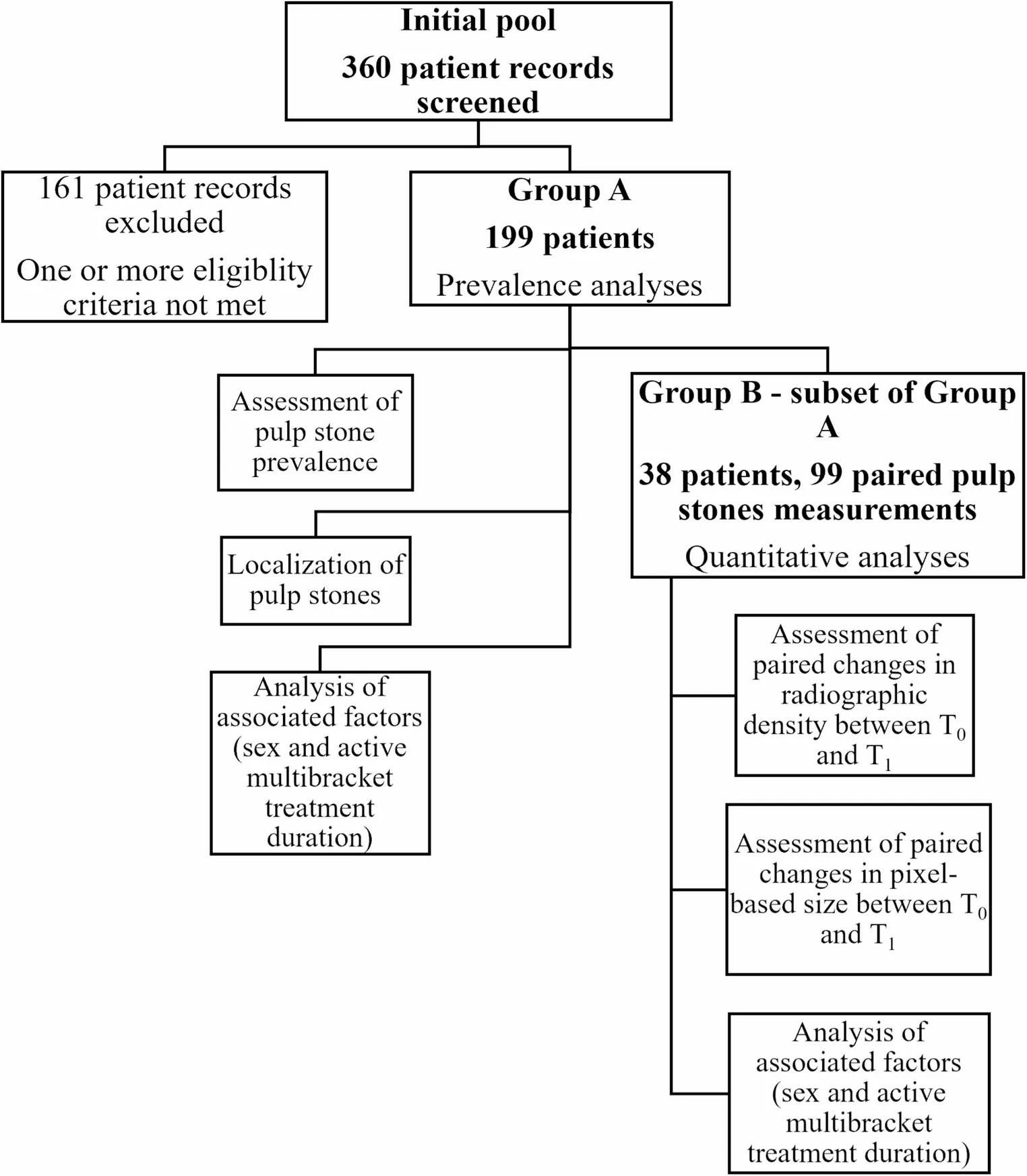
Schematic overview of patient screening, study design, and group allocation

The study population was divided into two analytical groups according to the respective outcomes. Group A comprised the entire study cohort (*n* = 199) and was used to assess longitudinal changes in pulp stone prevalence, pulp stone localization, and potentially associated factors (Fig. 1). Patient-level analyses evaluated prevalence changes and associated factors, whereas tooth-level analyses descriptively characterized the distribution and localization of pulp stones.

Group B comprised 38 patients with a total of 99 pre-existing pulp stones that could be identified and quantitatively assessed at both T_0_ and T_1_. This group was used to evaluate paired changes in the radiographic density and pixel-based size of the same pulp stones over time. Pulp stones newly detected at T_1_ were excluded from these quantitative analyses because no corresponding baseline measurement was available. Paired changes were summarized at the pulp-stone level, whereas analyses of potential associations with sex and active multibracket treatment duration were based on the mean change across all evaluable pulp stones within each patient (Fig. 1).

### Ethical considerations

This retrospective study exclusively used existing clinical data and radiographs obtained during routine orthodontic care. No additional intervention or radiographic examination was performed for research purposes, and all data were anonymized prior to scientific analysis. According to the recommendations of the Datenschutz und IT-Sicherheit im Gesundheitswesen (DIG) task force of the German Association for Medical Informatics, Biometry, and Epidemiology (GMDS), individual informed consent was not required for the analysis of anonymized routine-care data. Formal ethics committee approval was not obtained for this retrospective analysis.

### Clinical procedure

Fixed multibracket treatment followed a general clinical protocol, with individual modifications according to the respective clinical indication.

Experience Metal brackets and tubes with an MBT prescription and a 0.022-inch slot (GC Orthodontics Europe GmbH, Breckerfeld, Germany) were bonded in both arches from second molar to second molar. Bracket bonding was performed using Transbond XT (3 M Deutschland GmbH, Neuss, Germany). Treatment was carried out using a standardized archwire sequence consisting of thermoelastic nickel-titanium and stainless steel archwires (G&H Orthodontics Wire Company, Franklin, Indiana, USA).

Depending on individual indications, anterior or occlusal bite planes were applied. When clinically indicated, patients received instructions for the use of intra- and intermaxillary elastics. In approximately 67% of patients, multibracket treatment was preceded by functional orthodontic therapy (e.g., Twin Block, Activator). In approximately 8% of patients, additional appliances such as rapid palatal expanders and transverse developers (Ortho Organizers GmbH, Lindenberg, Germany) were used. Information on functional orthodontic pretreatment and the use of additional appliances was recorded only dichotomously. Detailed information regarding appliance type, duration of use, force magnitude, and the resulting tooth movements was not consistently available. Because these variables comprised heterogeneous interventions that could not be characterized in sufficient detail, they were not included as predictors in the final inferential models reported in this manuscript.

Panoramic radiographs at time point T_0_ were obtained as part of the initial diagnostic assessment prior to the initiation of any orthodontic treatment. Consequently, both functional orthodontic pretreatment and the use of additional appliances were initiated only after acquisition of the T_0_ panoramic radiographs.

Panoramic radiographs at time point T_1_ were obtained as part of the final diagnostic assessment after completion of the retention phase, approximately one year after removal of the multibracket appliances. The exact interval between removal of the multibracket appliances and acquisition of the T_1_ panoramic radiographs varied among patients and was not recorded separately in this retrospective analysis.

### Radiographic evaluation

All panoramic radiographs were acquired using the same radiographic device (Orthophos XG5, Sirona Dental Systems GmbH, Bensheim, Germany) with Sidexis XG software (version 2.61). Standardized acquisition parameters were used for all radiographs: an exposure time of 14.1 s, a tube voltage of 64 kV, and a tube current of 8 mA. The digital radiographic system was equipped with a detector with a pixel size of 27 μm. All visual and quantitative image assessments were performed by a single examiner using a calibrated diagnostic monitor (Radiforce MX216-SB, EIZO Corporation, Hakusan, Japan). The examiner was aware of the imaging time point and was therefore not blinded to whether a radiograph had been obtained at T_0_ or T_1_. The original images stored in the Sidexis database were available in a proprietary TIFF format with pixel dimensions of 2440 × 1280 pixels. The visual and quantitative evaluation procedures are described below.

For further quantitative analysis, the panoramic radiographs were exported from the proprietary TIFF format of the acquisition software to JPEG format. During export, a 12-pixel footer located outside the diagnostic image area was added, increasing the image dimensions from 2440 × 1280 to 2440 × 1292 pixels. The diagnostic image area retained its original pixel dimensions of 2440 × 1280 pixels, and no rescaling or geometric modification occurred. All ImageJ measurements were performed within manually delineated regions of interest located entirely within the diagnostic image area; therefore, the additional footer did not affect the pixel-based size or MGV measurements. Beyond this standardized export procedure, no additional image post-processing was performed.

**Fig. 2:**
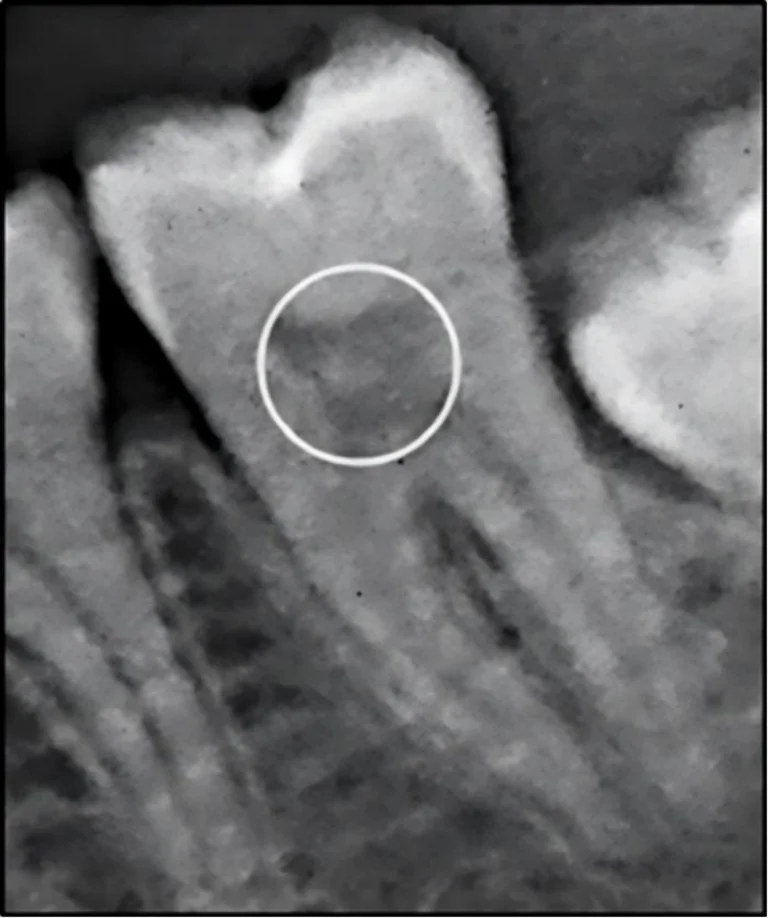
Pulp stone-positive molar

In Group A, pulp stone presence was assessed visually. To assess intrarater reliability, the visual assessment was repeated in 96 of the 199 patients after an interval of approximately four months. A tooth was classified as pulp stone-positive when a discrete, dense radiopaque structure was visible within the pulp chamber, consistent with previously applied radiographic diagnostic criteria [7, 25]. Only structures that could be clearly distinguished from the surrounding dental tissues and adjacent anatomical structures were scored as positive (Fig. 2).

**Fig. 3:**
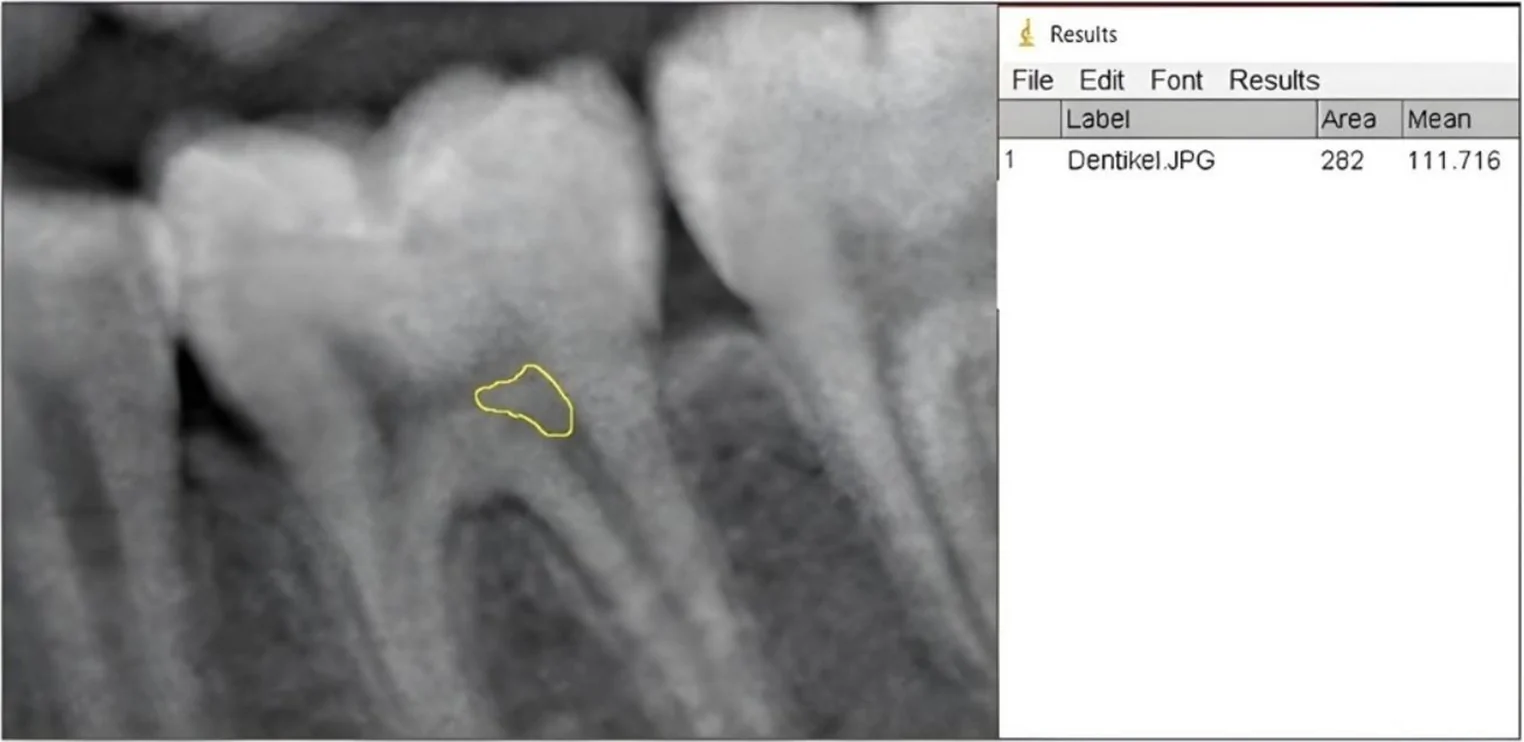
Quantitative assessment of a pulp stone in the mandibular left first molar (tooth 36) using ImageJ software

Findings in Group B were analyzed using ImageJ software (National Institutes of Health, Bethesda, Maryland, USA). The margins of each pulp stone were manually delineated, and the number of enclosed pixels was recorded as a pixel-based measure of pulp stone size (Fig. 3). The mean grey value (MGV) within the delineated area was calculated as a measure of radiographic density. Higher MGVs indicated greater radiopacity under the applied imaging and export conditions. Changes in MGV between T_0_ and T_1_ were used as an indirect radiographic surrogate for changes in calcification degree; however, MGV does not constitute a direct measurement of mineral content or composition. Changes in the measured parameters between T_0_ and T_1_ were subsequently calculated. To assess intrarater reliability, pixel-based size measurements were repeated for 80 pulp stones and MGV measurements for 68 pulp stones by the same examiner after an interval of approximately four months.

For quantitative analysis, only pulp stones that were clearly delineable on panoramic radiographs were included. Pulp stones with indistinct morphology, superimposition, or insufficient image quality were excluded from further analysis.

### Statistical analysis

Statistical analyses were performed using R software (version 4.1.1; R Core Team, R Foundation for Statistical Computing, Vienna, Austria) within the RStudio environment [31]. The level of statistical significance was set at *p* = 0.05 [32].

#### Intrarater reliability

In Group A, the visual assessment of pulp stone presence on the pretreatment (T_0_) and post-retention radiographs (T_1_) was repeated in 96 of the 199 patients. Agreement between the repeated dichotomous assessments (pulp stone present or absent) was evaluated using Cohen’s kappa coefficient.

In Group B, pixel-based size measurements were repeated for 80 pulp stones, and radiographic density measurements expressed as MGV were repeated for 68 pulp stones. Intrarater reliability of the continuous measurements was assessed using the intraclass correlation coefficient, ICC(3,1). In addition, Bland-Altman plots were generated to illustrate agreement and the distribution of differences between the repeated measurements.

Cohen’s kappa and ICC values were interpreted according to the criteria proposed by Landis and Koch [33] and Koo and Li [34], respectively.

#### Pulp stone prevalence and change in the number of pulp stone-positive molars (Group A; *n* = 199)

##### Patient-level analysis

Patient-level prevalence at T_0_ and T_1_ was compared using McNemar’s test. Sex-related differences in the transition from a negative status at T_0_ to a positive status at T_1_ were analyzed using the Pearson chi-square test. For analyses of associated factors, patient-level pulp stone increase was defined separately as an increase in the number of pulp stone-positive molars between T_0_ and T_1_, irrespective of the patient’s dichotomous prevalence status at baseline. Sex-related differences in this outcome were also analyzed using the Pearson chi-square test. Active multibracket treatment duration was assessed for normality using the Shapiro-Wilk test. Because it was not normally distributed, active multibracket treatment duration was compared between patients with and without an increase in the number of pulp stone-positive molars using the Mann-Whitney U test. To evaluate the associations of sex and active multibracket treatment duration with this latter patient-level outcome, a generalized linear model with a binomial error distribution and logit link function was applied. Model assumptions and potentially influential observations were assessed using standard regression diagnostics, including examination of multicollinearity and residuals. A sensitivity analysis was performed after excluding observations identified as potentially influential based on Cook’s distance.

##### Tooth-level analysis

At the tooth level, pulp stone occurrence at T_0_ and T_1_ was summarized descriptively for each evaluated tooth position to characterize the distribution and localization of the observed changes. No inferential tooth-level analysis was performed because multiple teeth originated from the same patient and therefore could not be considered statistically independent.

#### Quantitative analysis of radiographic density and pulp stone size (Group B; *n* = 38)

##### Radiographic density

Changes in mean grey value (MGV) between T_0_ and T_1_ were calculated for each of the 99 pre-existing pulp stones and summarized descriptively at the pulp-stone level. MGV change was interpreted as an indirect imaging-based surrogate for change in calcification degree rather than as a direct measure of mineral content. For analyses of associated factors, the mean MGV change across all evaluable pulp stones within each patient was calculated. Sex-related differences in patient-level mean MGV change were analyzed using Welch’s independent-samples t-test after assessment of normality. Active multibracket treatment duration was assessed for normality using the Shapiro-Wilk test, and its association with patient-level mean MGV change was evaluated using Spearman’s rank correlation. A multiple linear regression model including sex and active multibracket treatment duration as predictors was used to further explore these associations.

##### Pulp stone size

Changes in pixel-based size between T_0_ and T_1_ were calculated for each of the 99 pre-existing pulp stones and summarized descriptively at the pulp-stone level. For analyses of associated factors, the mean pixel-based size change across all evaluable pulp stones within each patient was calculated. Sex-related differences in patient-level mean size change were analyzed using Welch’s independent-samples t-test after assessment of normality. Active multibracket treatment duration was assessed for normality using the Shapiro-Wilk test, and its association with patient-level mean size change was evaluated using Spearman’s rank correlation. A multiple linear regression model including sex and active multibracket treatment duration as predictors was used to further explore these associations. For both multiple linear regression models, model assumptions and potentially influential observations were assessed using standard regression diagnostics, including examination of residual distributions and multicollinearity. Sensitivity analyses were performed after excluding observations identified as potentially influential based on Cook’s distance.

## Results

### Intrarater reliability (Groups A and B)

**Fig. 4:**
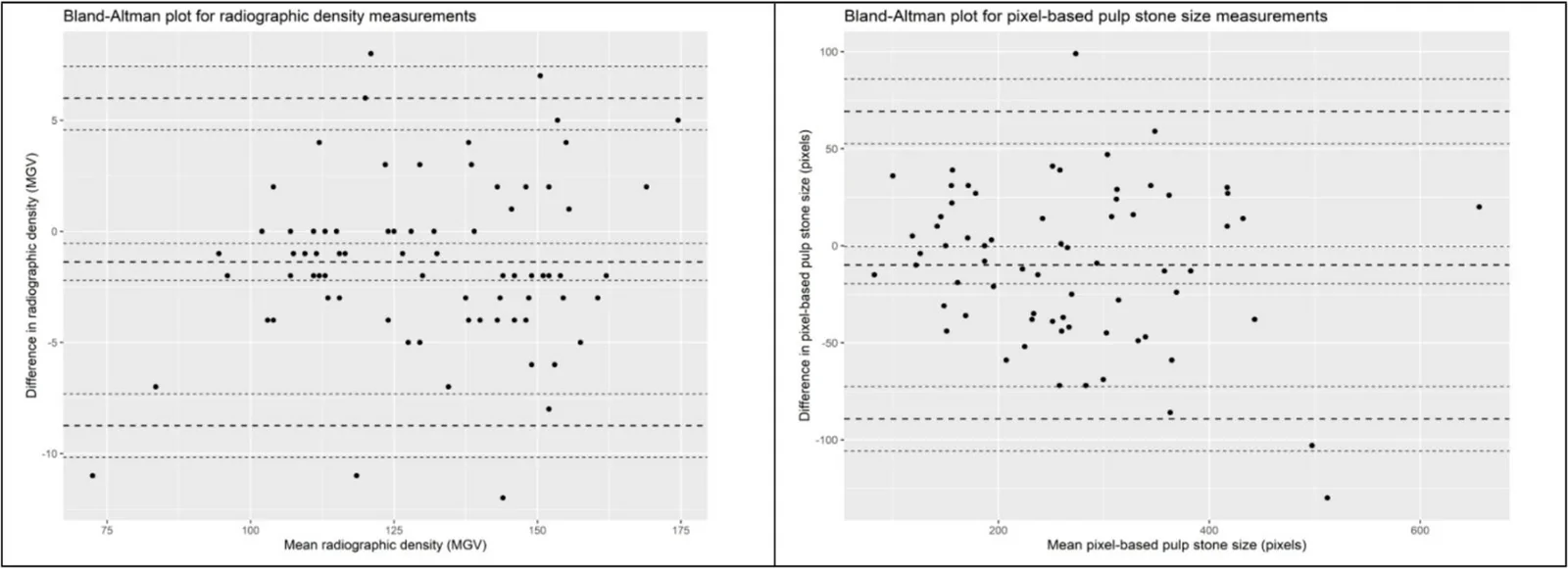
Bland-Altman plots for radiographic density expressed as mean grey value (left) and pixel-based pulp stone size (right)

The repeated assessments demonstrated almost perfect intrarater agreement for the visual assessment and excellent intrarater reliability for both quantitative measurements. In Group A (*n* = 96), the repeated visual assessments yielded a Cohen’s kappa coefficient of κ = 0.87, indicating almost perfect agreement. For the repeated measurements of radiographic density, the ICC(3,1) was 0.98 (*n* = 68), while the ICC(3,1) for pixel-based pulp stone size was 0.93 (*n* = 80); both values indicate excellent reliability (Table 1). Bland-Altman plots showed a largely uniform distribution of measurement differences for both parameters without an indication of systematic bias, with most values located within the limits of agreement (Fig. 4).

**Table 1:** Results of intrarater reliability assessment (Groups A and B)

Group	Assessment	Sample size (*n*)	Parameter		Statistic	Value
A	Pulp stone presence (T_0_ and T_1_)	96		Cohen’s kappa	κ	0.87
B	Radiographic density (MGV)	68		Intraclass correlation coefficient	ICC(3,1)	0.98
B	Pixel-based pulp stone size	80		Intraclass correlation coefficient	ICC(3,1)	0.93

### Group A (*n* = 199)

#### Pulp Stone Occurrence

Patient-level analyses describe the proportion of patients with at least one radiographically detectable pulp stone and the transitions in individual patient status between T0 and T1. Tooth-level analyses describe the proportion and distribution of affected teeth among the 1,592 evaluated molars and were performed descriptively because multiple teeth originated from the same patient.

Patient-level analysis: Pulp stone prevalence increased by 9.0% points, from 19.6% at T_0_ to 28.6% at T_1_. McNemar’s test revealed a statistically significant difference in prevalence between the two time points (*p* < 0.001) (Table 2). No patient transitioned from pulp stone-positive at T_0_ to pulp stone-negative at T_1_; all 39 patients with pulp stones at baseline remained pulp stone-positive at follow-up.

**Table 2:** Overall patient-level pulp stone prevalence and transition from T_0_ to T_1_ (Group A)

Time point	Pulp stone positive *n* (%)	Pulp Stone negative *n* (%)	Total (*n*)
T_0_	39 (19.6%)	160 (80.4%)	199
T_1_	57 (28.6%)	142 (71.4%)	199

Transition from T_0_ to T_1_			
T_0_ → T_1_	Negative T_1_	Positive T_1_	
Negative at T_0_	142	18	
Positive at T_0_	0	39	

McNemar’s test: *p* < 0.001

At T_0_, pulp stone prevalence was similar in females and males. At T_1_, prevalence was higher in males, corresponding to an increase of 17.1% points in males compared with 3.4% points in females, and the difference in prevalence changes between the sexes was statistically significant (*p* = 0.001; Table 3). The proportion of patients showing an increase in the number of pulp stone-positive molars was also significantly higher among males (*p* = 0.04; Table 3).

**Table 3:** Sex-specific prevalence and increase in the number of pulp stone-positive molars (Group A)

Prevalence at T_0_ and T_1_				
Sex	T_0_ positive *n* (%)	T_1_ positive *n* (%)	Increase *n* (percentage points)	*p* value
Male (*n* = 82)	16 (19.5%)	30 (36.6%)	14 (17.1)	0.001
Female (*n* = 117)	23 (19.7%)	27 (23.1%)	4 (3.4)	

Increase in the number of pulp stone-positive molars from T_0_ to T_1_				
Sex	No increase n (%)	Increase n (%)		p value
Male (*n* = 82)	57 (69.5%)	25 (30.5%)		0.04
Female (*n* = 117)	96 (82.1%)	21 (17.9%)		
p values were derived from Pearson chi-square tests for between-sex comparisons				

Mean active multibracket treatment duration did not differ between patients with an increase in the number of pulp stone-positive molars (16.76 ± 4.42 months) and those without an increase (16.61 ± 3.75 months). The Mann-Whitney U test showed no statistically significant difference between the groups (*p* = 0.90).

Logistic regression analysis showed a significant association between sex and an increase in the number of pulp stone-positive molars per patient, whereas active multibracket treatment duration was not statistically associated with the outcome (Table 4). Regression diagnostics did not indicate substantial violations of model assumptions, and a sensitivity analysis excluding potentially influential observations did not alter the conclusions of the logistic regression model.

**Table 4:** Logistic regression analysis of factors associated with an increase in the number of pulp stone-positive molars per patient (Group A)

Variable	Estimate	Standard error	z value	*p* value	Odds ratio	2.5%	97.5%	VIF
Intercept	-0.908	0.775	-1.17	0.241	0.403	0.088	1.869	
Sex	-0.693	0.341	-2.04	0.042	0.500	0.255	0.972	1.00
Active treatment duration	0.005	0.043	0.11	0.909	1.005	0.921	1.093	1.00

The outcome was an increase in the number of pulp stone-positive molars within a patient between T_0_ and T_1_ (0 = no increase; 1 = increase). The model included sex and active multibracket treatment duration as predictors. Male sex was the reference category, and active multibracket treatment duration was measured in months

*SE* standard error, *VIF* variance inflation factor

At the tooth level, pulp stone prevalence increased between T_0_ and T_1_ in all evaluated first and second molars. Maxillary molars showed higher prevalence at both time points, whereas the largest percentage-point increase was observed in the mandibular left first molar (tooth 36; Table 5).

**Table 5:** Tooth-level changes in pulp stone prevalence from T_0_ to T_1_ (Group A)

Tooth	T_0_*n* (%)	T_1_*n* (%)	Δn (absolute increase)	Δ (percentage points)
17	5 (2.5%)	16 (8.0%)	+ 11	+ 5.5
16	31 (15.6%)	43 (21.6%)	+ 12	+ 6.0
26	24 (12.1%)	38 (19.1%)	+ 14	+ 7.0
27	9 (4.5%)	21 (10.6%)	+ 12	+ 6.0
37	4 (2.0%)	11 (5.5%)	+ 7	+ 3.5
36	16 (8.0%)	35 (17.6%)	+ 19	+ 9.5
46	13 (6.5%)	28 (14.0%)	+ 15	+ 7.5
47	2 (1.0%)	9 (4.5%)	+ 7	+ 3.5

Teeth were designated according to the FDI two-digit notation: the first digit indicates the quadrant (1, maxillary right; 2, maxillary left; 3, mandibular left; 4, mandibular right), and the second digit indicates the tooth type (6, first molar; 7, second molar)

### Group B (*n* = 38)

#### Radiographic Density

Pooled pulp-stone-level descriptive analyses indicated a positive mean change in radiographic density, although substantial variability was observed among the 99 individual pulp stones. At the patient level, mean MGV changes did not differ significantly according to sex (*p* = 0.79) and were not significantly correlated with active multibracket treatment duration (Spearman’s ρ = -0.18, *p* = 0.275). In the multiple linear regression model, neither sex (*p* = 0.845) nor active multibracket treatment duration (*p* = 0.095) was significantly associated with patient-level mean MGV change. The overall model was not statistically significant and showed minimal explanatory value (*p* = 0.235; Table 6). Regression diagnostics did not indicate substantial violations of model assumptions, and exclusion of two potentially influential observations in a sensitivity analysis did not alter the conclusions.

#### Pulp Stone Size

Pooled pulp-stone-level descriptive analyses indicated a positive mean change in pixel-based pulp stone size, although substantial variability was observed among the 99 individual pulp stones. At the patient level, mean pixel-based size changes did not differ significantly according to sex (*p* = 0.93). Active multibracket treatment duration was not significantly correlated with patient-level mean size change in the Spearman analysis (ρ = 0.14, *p* = 0.387). In the multiple linear regression model, active multibracket treatment duration was positively associated with patient-level mean size change (*p* = 0.034), whereas sex was not significantly associated with the outcome (*p* = 0.626). However, the overall regression model was not statistically significant and explained only a small proportion of the observed variability (*p* = 0.102; Table 6). Three potentially influential observations were identified in the pixel-based size model. After their exclusion, the association with active multibracket treatment duration was attenuated and no longer statistically significant (*p* = 0.105), consistent with the non-significant Spearman correlation.

**Table 6:** Changes in radiographic density and pixel-based pulp stone size between T_0_ and T_1_ (Group B)

A. Overall changes					
Parameter	Mean change	SD			
Radiographic density (MGV)	6.0	16.0			
Pixel-based pulp stone size	62.5	124.8			

B. Sex-related differences					
Parameter	Male mean ± SD	Female mean ± SD	p value		
Mean MGV change	5.74 ± 9.86	6.59 ± 9.45	0.79		
Mean pixel-based size change	54.92 ± 125.42	51.70 ± 65.23	0.93		

C. Linear regression models					
Model 1 – Changes in radiographic density (MGV)					
Variable	Estimate	SE	t	p value	VIF
Intercept	17.45	7.22	2.42	0.021	-
Sex	-0.63	3.20	-0.20	0.845	1.08
Active treatment duration	-0.67	0.39	-1.72	0.095	1.08
Adjusted R² = 0.027; F(2, 35) = 1.51; *p* = 0.235					

Model 2 – Changes in pixel-based pulp stone size					
Variable	Estimate	SE	t	p value	VIF
Intercept	-90.21	69.52	-1.30	0.203	-
Sex	15.18	30.82	0.49	0.626	1.08
Active treatment duration	8.32	3.77	2.21	0.034	1.08
Adjusted R² = 0.072; F(2, 35) = 2.44; *p* = 0.102					

Descriptive values were pooled across 99 paired measurements of pre-existing pulp stones from 38 patients. Sex comparisons and linear regression models used patient-level mean changes (*n* = 38). Male sex was the reference category; active treatment duration was measured in months

*MGV* mean grey value, *SD* standard deviation, *SE* standard error, *VIF* variance inflation factor

## Discussion

The aim of this retrospective study was to characterize longitudinal changes in pulp stone prevalence and to explore paired changes in the radiographic density and pixel-based size of pre-existing pulp stones in patients undergoing fixed orthodontic treatment. Potential associations with sex and active multibracket treatment duration were also examined.

### Methodological considerations

The restriction of the study population to adolescents limits the generalizability of the findings because pulp stone prevalence increases with age [13]. Local dental conditions, including caries and restorations, have been investigated as potential correlates of pulp stone formation [35–40]. Possible associations with systemic diseases have also been investigated, although the findings remain inconsistent [41–49]. To reduce potential confounding, patients with known systemic diseases and teeth affected by caries, extensive restorations, or previous endodontic treatment were excluded.

Digital panoramic radiographs were used for pulp stone detection, consistent with previous orthodontic studies [7–12]. They were routinely available at both observation times and enabled longitudinal assessment without additional radiation exposure. However, their limited spatial resolution, two-dimensional projection, and anatomical superimposition may impair the detection and localization of small or radicularly located calcifications and may also result in uncertain radiopaque findings [26, 27, 50]. Chen et al. detected more pulp stones using periapical than panoramic radiographs [26], while comparison with CBCT demonstrated the potential for superimposition-related misinterpretation on panoramic images [27]. Prevalence estimates should therefore be interpreted in relation to the imaging modality used.

ImageJ was used to perform paired measurements of pixel-based size and mean grey value in pre-existing pulp stones. However, geometric distortion, variable magnification, patient positioning, and image-processing conditions affect measurements obtained from panoramic radiographs [26, 27]. Pixel-based measurements do not represent true physical area or three-dimensional volume, and uncalibrated MGV cannot be interpreted as an absolute density value or a direct measure of mineral content. Manual delineation is examiner-dependent, and the inclusion of only clearly delineable pulp stones may have introduced selection bias. The quantitative analyses should therefore be regarded as exploratory.

High intrarater reliability and no indication of systematic bias in the Bland-Altman analyses support repeatability under the applied conditions but do not establish external validity or absolute accuracy. Interrater reliability was not assessed. Future validation should incorporate calibrated reference standards and multiple blinded examiners; higher-resolution or three-dimensional imaging may also be considered where clinically justified.

### Prevalence

The reported prevalence of pulp stones varies considerably, with older literature reporting values ranging from approximately 8% to 90% [23, 24]. More recently, Kaabi et al. detected pulp stones in 98.3% of the examined dental arches and 52.1% of the evaluated teeth using CBCT [28]. These estimates are not directly comparable with patient- or tooth-level prevalence derived from panoramic radiographs because the study used three-dimensional imaging, included the entire coronal and radicular pulp space, and examined an adult Saudi population [28]. In the present study, patient-level prevalence increased by 9.0% points, from 19.6% at T_0_ to 28.6% at T_1_ (*p* < 0.001), while descriptive increases were also observed at the tooth level in both jaws.

Previous orthodontic studies have generally reported increases in pulp stone occurrence, although their magnitude and statistical significance have varied [7–12]. Significant increases were reported in several studies [7–10], whereas Sarang et al. observed only a slight, non-significant increase [11]. More recently, Ba-Hattab et al. reported increases among patients treated with fixed appliances or clear aligners but found no significant associations with appliance type, force application, or active treatment duration [29]. Gholinezhad et al. similarly observed significant increases in both extraction and non-extraction groups undergoing fixed orthodontic treatment, without a significant difference between the treatment protocols [30]. These findings are consistent with the direction of change observed in the present cohort but suggest that the reported increases may not be specific to a particular appliance or extraction protocol.

Direct comparison among studies remains limited by differences in study design, imaging modality, evaluated pulpal regions and tooth types, units of analysis, age distribution, geographic setting, treatment protocol, observation period, and inclusion criteria [7–12, 23, 25–28]. For example, Sarang et al. examined only premolars [11], whereas the present study was restricted to first and second molars, which are more frequently affected by pulp stones [7, 35, 51, 52]. Differences among panoramic, periapical, and CBCT imaging may additionally influence detection because of differences in spatial resolution, anatomical representation, and superimposition-related misinterpretation [26, 27]. Thus, although the direction of change in the present cohort is broadly consistent with the available literature, direct comparison of prevalence estimates and effect magnitudes remains limited.

### Sex

No sex-specific difference in pulp stone prevalence was observed at T_0_, consistent with previous studies [35, 36, 52, 53]. However, the increase between T_0_ and T_1_ was significantly greater in males than in females (17.1 versus 3.4% points). A numerically greater increase in males was also reported by Jena et al. [10] but contrasts with Afsari et al., who observed significant post-treatment changes only among females [9], while other orthodontic studies found no significant sex-related differences [7, 8, 11]. The available evidence therefore does not support an established explanation for the observed association. Because biological, developmental, and treatment-related explanatory variables were not assessed, any mechanistic interpretation would be speculative. The sex-related association should be regarded as exploratory and requires confirmation in controlled studies.

### Active multibracket treatment duration

Active multibracket treatment duration was not significantly associated with an increase in the number of pulp stone-positive molars per patient. The overall mean of 16.6 months was shorter than the average duration of approximately 20 months reported for fixed orthodontic treatment [54]. This may partly reflect the restriction of the present cohort to non-extraction cases without combined orthodontic-orthognathic surgical treatment.

Previous findings regarding treatment duration are inconsistent. Babanouri et al. reported a significant association, particularly for treatment periods exceeding 31 months [7], whereas Kublitski et al. and Ba-Hattab et al. found no significant association [29, 55]. Differences in treatment duration, appliance protocols, extraction status, and evaluated tooth types limit direct comparison among studies. The present finding should therefore be interpreted in the context of the comparatively short and homogeneous active treatment periods and should not be extrapolated to longer or more complex treatment courses.

### Localization

Pulp stones were most frequently detected in first molars and maxillary molars, consistent with previous studies [8, 10, 36, 40, 42, 51, 52, 56–60]. Maxillary molars showed higher prevalence at both time points, whereas the descriptive magnitude of change was broadly comparable between the jaws. No clear descriptive difference was observed between the left and right sides. Several studies likewise reported no lateral difference [43, 45, 60], whereas others found a higher prevalence on the left side [42, 46]. The available evidence regarding lateral distribution therefore remains inconsistent.

### Radiographic density

A positive pooled pulp-stone-level mean change in MGV was observed between T_0_ and T_1_, although substantial variability occurred among individual measurements. Neither sex nor active multibracket treatment duration was significantly associated with patient-level mean MGV change. Because MGV was not calibrated against a radiographic reference standard, the observed change cannot directly demonstrate progressive mineralization or altered mineral content. Histological and material-analytical studies describing structural and chemical similarities between pulp stones and dentin provide a biologically plausible context but do not establish the mechanism underlying the present imaging-based findings [61, 62]. Although pulpal reactions to orthodontic forces have been reported, the available evidence remains heterogeneous [16, 17]. The present analysis cannot determine whether the observed MGV changes reflect biological alterations, imaging-related variability, or both. Further studies using calibrated imaging, higher-resolution techniques, or histological analyses are required.

### Pulp stone size

A positive pooled pulp-stone-level mean change in pixel-based pulp stone size was observed between T_0_ and T_1_, with substantial variability among individual measurements. The most pronounced descriptive changes occurred in mandibular molars. Progressive deposition of a mineralized matrix could plausibly contribute to both increased radiographic density and an apparent increase in two-dimensional size [61–63]. However, the present imaging-based findings cannot demonstrate the underlying biological or mineralization processes, and direct comparative studies of longitudinal pulp stone size changes are lacking. These findings should therefore be regarded as exploratory.

The association between active multibracket treatment duration and pixel-based size change was inconsistent. Although active multibracket treatment duration had a statistically significant individual coefficient in the multiple linear regression model, the overall model was not statistically significant, explained only a small proportion of the variability, and was not supported by Spearman’s rank correlation. Moreover, the association was attenuated and no longer statistically significant after exclusion of three potentially influential observations in the sensitivity analysis. Given the small sample size of Group B and the substantial measurement variability, the coefficient does not constitute robust evidence of a treatment duration-dependent change in pixel-based pulp stone size.

Overall, the cautious interpretation of the present findings is consistent with a recent systematic review, which concluded that orthodontic treatment may modestly increase dental pulp calcifications, particularly pulp stones, but rated the certainty of evidence as low to very low because of substantial clinical and methodological heterogeneity [64]. The review also emphasized that clinical relevance remains uncertain and that high-quality prospective studies are needed [64].

### Clinical implications

The present findings do not justify changes to established orthodontic treatment protocols or additional radiographic examinations solely for the detection of pulp stones. Nevertheless, pulp stones identified incidentally on routinely indicated orthodontic radiographs may be documented. Although pulp stones generally do not require treatment in the absence of additional clinical signs or symptoms, larger or strategically located calcifications may alter the internal anatomy of the pulp chamber, obstruct access to canal orifices, or impede root canal instrumentation [13]. Awareness of pre-existing or newly detectable pulp stones may therefore be relevant if endodontic treatment becomes necessary later. Any further radiographic assessment should be based on an individual clinical indication and the principles of radiation protection.

### Limitations

A principal limitation is the absence of an untreated control group. Consequently, the observed increase in pulp stone prevalence cannot be attributed exclusively to fixed orthodontic treatment. Age-related changes, elapsed observation time, post-treatment maturation, and other unmeasured factors may have contributed. The findings should therefore be interpreted as longitudinal changes within an orthodontically treated cohort rather than as evidence of a causal treatment effect. Future controlled studies with age-matched untreated participants and standardized observation intervals are required to distinguish treatment-associated from naturally occurring changes.

A further limitation is the heterogeneity of the orthodontic interventions. After T_0_ imaging, approximately two thirds of the patients underwent functional orthodontic pretreatment, and approximately 8% received additional expansion or transverse-development appliances. These interventions may have exerted additional mechanical forces on the teeth and pulpal tissues. However, they were recorded only dichotomously, while detailed information on appliance type, duration, force magnitude, and resulting tooth movements was unavailable. Their potential contribution could therefore not be evaluated reliably or separated from that of fixed appliances. This represents a potential source of confounding.

Systemic and medication-related factors should also be considered. Although patients with known systemic diseases were excluded, chronic medication use was not evaluated independently. Associations between pulp stones, systemic diseases, and chronic medication use have been reported in unerupted teeth, suggesting that pulpal calcifications are not necessarily dependent on local oral or orthodontic influences [15]. Residual confounding by unrecorded systemic or medication-related factors therefore cannot be excluded.

The timing of T_1_ imaging also limits temporal interpretation. T_1_ radiographs were obtained after the retention phase, typically about one year after removal of the fixed appliances, while the exact post-debonding interval varied and was not recorded separately. Consequently, the observed changes encompass both active multibracket treatment and the subsequent retention period and cannot be assigned to either phase. Moreover, the recorded treatment duration represented only active multibracket treatment rather than the entire observation interval between T_0_ and T_1_. Future studies should use standardized imaging intervals to permit more precise temporal characterization of pulp stone changes.

The examiner was not blinded to the imaging time point, which may have introduced observer bias. Although predefined diagnostic criteria and repeated assessments demonstrated high intrarater reliability, they do not eliminate this potential source of bias.

Reason-specific exclusion counts were not retained in the retrospective screening documentation. Consequently, the relative contribution of individual eligibility criteria to the exclusion of the 161 patient records could not be reconstructed, limiting detailed assessment of the selection process.

The single-center setting in a private orthodontic practice in northern Germany limits generalizability to populations with different demographic, geographic, or healthcare-related characteristics. Data on individual nationality and ethnic background were unavailable and could therefore not be considered.

No formal a priori sample size calculation was performed. Consequently, non-significant associations should be interpreted cautiously because limited statistical power cannot be excluded, particularly for the exploratory quantitative analyses in Group B (*n* = 38).

## Conclusion

In this retrospective cohort of orthodontically treated patients, patient-level pulp stone prevalence increased significantly between the pretreatment and post-retention radiographs, with a greater increase observed in males than in females. Active treatment duration was not significantly associated with an increase in the number of pulp stone-positive molars per patient. At the tooth level, descriptive increases were observed in all evaluated first and second molars, while maxillary molars showed higher prevalence at both time points.

Exploratory quantitative analyses indicated positive mean changes in the radiographic density and pixel-based size of pre-existing pulp stones, with no sex-related differences. Findings regarding the association between active treatment duration and pixel-based size change were inconsistent.

These findings describe longitudinal changes in an orthodontically treated cohort but do not establish that fixed orthodontic treatment caused these changes. Future controlled longitudinal studies including age-matched untreated comparison groups and standardized observation intervals are needed to distinguish treatment-associated changes from naturally occurring changes over time.

## Data Availability

The datasets generated and/or analyzed during the current study are not publicly available due to data protection and privacy regulations but are available from the corresponding author on reasonable request.
